# Deep breast pain during lactation: a case-control study in Sweden investigating the role of *Candida albicans*

**DOI:** 10.1186/s13006-018-0167-8

**Published:** 2018-06-07

**Authors:** Kirsti Kaski, Linda J. Kvist

**Affiliations:** 1 Deparment for Obstetrics & Gynaecology, Helsingborg Hospital, 25187 Helsingborg, Sweden; 2 Health Sciences Centre, Faculty of Medicine, Lund University, Box 157, 221 00 Lund, Sweden

**Keywords:** Breast milk, *Candida albicans*, Deep breast pain, Breastfeeding, Breastfeeding self-efficacy

## Abstract

**Background:**

Deep breast pain during lactation, with or without accompanying nipple pain and soreness continues to be anecdotally linked to infection by *Candia albicans* despite lack of robust evidence in the literature that *Candida albicans* is the cause of women’s breast symptoms.

**Methods:**

A case-control study of breastfeeding women in Sweden with (*n* 35) and without (*n* 35) symptoms that may be attributable to *Candida albicans* was carried out. The symptoms were radiating, burning and penetrating or non-penetrating breast pain with or without associated nipple pain during or after breastfeeding. The primary aim of the study was to test the hypothesis that breastfeeding women with symptoms commonly associated with *Candida albicans* infection will have a growth of *Candida albicans* in their breast milk significantly more often than women without these symptoms. A secondary aim was comparison of breastfeeding self-efficacy, measured by the BSES-SF (Breastfeeding Self Efficacy Scale –Short Form), between cases and controls.

**Results:**

None of the women in the control group and eight of the women in the case group showed a growth of *Candida albicans* in their breast milk (*p* <  0.01), which confirms the hypothesis. There were no statistically significant differences in severity or type of symptoms between those in the case group with and without growth of *Candida albicans* in their breast milk. Results of the BSES-SF measurement showed no statistically significant differences between cases and controls. However, when analyses were stratified for parity, multiparous controls showed statistically significant higher scores for breastfeeding self-efficacy than multiparous cases.

**Conclusions:**

Neither clinical symptoms nor microbial cultivation appear to be reliable means for making a diagnosis of *Candida albicans* infection of the breast. Skilled breastfeeding consultants should offer support and help with positioning, attachment and identification of physical impediments to successful breastfeeding. Professionals should be aware that it is possible that uncertainty in the breastfeeding situation may to some extent account for mothers’ breast symptoms. The ISRCTN (International Standard Randomised Controlled Trial Number) identity for this case-control study is ISRCTN88839993. The study was retrospectively registered on 30 November 2016.

## Background

*Candida *species (spp) are part of the normal microbial flora of the human gut and *Candida albicans* is by far the most prevalent of the *Candida* species found in humans [1]. When opportunity arises, the organism can overgrow and result in an infectious process particularly in immuno-insufficient individuals. Breastfeeding practitioners commonly suggest *Candida albicans* as the cause of deep breast pain during lactation but despite attempts by researchers to clarify the role played by *Candida albicans*, uncertainty remains about the cause(s) of women’s breast symptoms [2–6]. Francis-Morrill et al. report the symptoms as radiating, burning and penetrating or non-penetrating breast and nipple pain during and after breastfeeding [4]. This pain may be accompanied by dry scaling skin on the nipple and areola, described as vivid pink, thinning or shiny [4].

Studies report findings in different ways: some report on women with symptoms of breast and nipple pain, others report on women with and without pain symptoms. In some studies, all *Candida* spp. are reported and in others only *Candida albicans.* Results show a variation between 2 and 23% for positive cultivation of *Candida albicans* or *Candida* spp. in breast milk samples from women both with and without symptoms [2–6]. An Australian cohort study of healthy breastfeeding mothers and their infants showed a growth of *Candida albicans* in the milk of 2.6% of all breastfeeding women (*n* = 346) during four postpartum weeks, whereas the results for any *Candida* spp. showed a growth in 5% of the cohort [2]. In a study from USA, breast milk samples were examined from 16 women with symptoms that are commonly said to be attributable to *Candida albicans* infection and from 18 women who did not complain of these symptoms [3]. A single colony of *Candida albicans* was identified in one of the 68 breast milk samples tested in the study (2.9% of all study women): the infected sample belonged to a woman with symptoms [3]. Francis-Morrill et al. also reported on a study from USA where 100 healthy breastfeeding women were included and showed that 23% of women had a positive culture for *Candida* spp. on either the nipple/areola or in breast milk [4]. This is in contrast to a USA study of similar size, where the authors found that 12% of all breastfeeding women in the study, with and without symptoms, had a growth of *Candida albicans* in their milk [5]. Differences in results from the studies may to some extent be caused by differences in measures used to limit contamination such as the use of routine breast cleansing procedures prior to culture collection.

In a recent study from Spain, researchers collected and examined breast milk from two groups of breastfeeding women [6]. The first group of 60 women included 30 with deep breast pain or nipple pain and 30 without pain. These women had sampled their breast milk by using their own personal breast pumps or by hand expression. The second group of 529 women all complained of deep breast pain and some also complained of nipple pain. These women sampled their milk by hand expression alone. The authors found *Candida* spp. to be more common in those who used their own breast pumps and suggested contamination as the cause of this. In the first group of 60 women 8.3% were found to have *Candida* spp. in their breast milk and in the second group of 529 women, *Candida* spp. were found in 2% of samples. Despite the reported difficulties in determining the origin of women’s symptoms, it has been shown that 93% of caregivers base their diagnosis of *Candida albicans* infection solely on the clinical picture [7]. Some researchers have suggested that bacterial infection might in part be responsible for the symptoms that have been attributed to *Candida albicans* [2, 3, 6] although others have shown that even breast milk from healthy women contains a myriad of bacteria, many of which may be considered as potential pathogens [8, 9].

### The complex process of breastfeeding

Breastfeeding is a complex process and its success is dependent on many internal and external factors. A woman’s previous life experiences, her attitudes and general self-confidence are examples of internal factors whereas socioeconomical factors, traditions, health service providers’ attitudes, family support and mother or infant health issues are examples of external factors [10–12]. One factor in particular, the belief in one’s capacity to breastfeed, has been scientifically evaluated. According to Dennis, breastfeeding self-efficacy is composed of two parts: the belief that a behaviour will produce a specific outcome and the conviction that oneself can successfully perform the behaviour in order to attain the desired outcome [13]. The Breastfeeding Self-Efficacy Scale (BFSES) is a validated instrument that measures breastfeeding self-confidence [13]. Amir et al. discuss how many factors can alter women’s experiences of breastfeeding pain [14]. Those who, for example, experience stress, anxiety, lack of social support and fatigue may also experience heightened sensitivity to breast pain during lactation [14]. Women with low breastfeeding self-confidence may need extra attention when experiencing breast pain during lactation [15].

Recent research has suggested that lifestyle and environmental factors affect the immunological composition of breast milk [16]. This in turn suggests that the milk microbiome also differs according to geographical and socioeconomic settings, which could possibly be one reason for disparate findings in studies on *Candida albicans* in different populations. The present study was designed to emulate previous research in order to add to the bank of knowledge by examining the occurrence of *Candida albicans* in a Swedish breastfeeding population.

The primary aim of this case-control study was to test the hypothesis that breastfeeding women with symptoms commonly associated with *Candida albicans* infection (cases) will have a growth of *Candida albicans* in their breast milk significantly more often than women without these symptoms (controls). A secondary aim was to compare breastfeeding self-efficacy scores between cases and controls.

## Methods

The cases were breastfeeding women with self-reported symptoms that are anecdotally related to *Candida albicans* infection of the breast: radiating, burning and penetrating or non-penetrating breast pain with or without associated nipple pain during or after breastfeeding. These symptoms may or may not be accompanied by vivid pink dry, scaling, thinning or shiny skin on the nipple and areola. The controls were breastfeeding women who did not have any of these symptoms. All infants were exclusively or partially breastfed, no limitation for the age of the infant was applied.

### Sample size calculation

The literature shows a wide variation in the presence of *Candida albicans* in breast milk and in order to allow a sample size calculation it was hypothesised, based on previous studies, that there should be a 33% higher occurrence of *Candida albicans* in the breast milk of women with symptoms traditionally associated with *Candida albicans* infection than in the breast milk from women without symptoms. Based on α = 0.05 and β = 0.2 with a difference of 33% between the groups the sample size was determined as 35 × 2.

### Study setting

The study took place at a breastfeeding clinic attached to an Obstetrics and Gynaecology Unit at a district hospital in southern Sweden. The breastfeeding clinic was started in 1992 and is situated in a town without particular socioeconomical problems. Today, four midwives with long experience of breastfeeding and its problems manage the clinic. Three of the four midwives have completed university courses on breastfeeding and two of the four have passed the IBCLC examination (International Board Certified Lactation Consultant). Breastfeeding women can contact the clinic for telephone guidance or to book a time for a consultation. In the hospital up-take area there are 46 well-baby clinics and of these, three took part in the study by identifying women without symptoms and who were prepared to take part in the study as the control group. The control group was also partially recruited from the hospital unit where postpartum consultations are carried out.

### Sample, recruitment and study population

For inclusion in either group women were required to be healthy, to understand spoken and written Swedish and to be currently breastfeeding either partially or exclusively. For inclusion in the case group women either consulted the breastfeeding clinic or were referred there by other care providers because of problems with radiating, burning and penetrating or non-penetrating breast pain with or without nipple pain during or after breastfeeding. These symptoms could be accompanied by pink dry, scaling, thinning or shiny skin on the nipple and areola but skin changes were not criteria for inclusion. Women who had pain or nipple damage that were diagnosed by the attending midwife as being caused by breastfeeding technique problems or mastitis were excluded, as were women who had skin symptoms that were diagnosed as eczema. Women in the control group were required to have no pain and normal nipple appearance.

The final sample consisted of 35 women in the case group and 35 women in the control group. A total of 11 women were excluded from participation in each of the groups because they did not meet the inclusion criteria, they refused to partake or there were logistic problems in finding appointment times that suited them.

### Material and data collection

Data were collected between March 2014 and March 2017. After informed consent had been acquired, each individual was asked questions about background variables; mothers’ and infant’s age, mother’s parity, type of birth, history of gestational diabetes or type 1 diabetes, history of vaginal *Candida albicans* or breast *Candida albicans* infections, previous breastfeeding and intake of antibiotics during or after birth. The women with symptoms were asked to localise their pain: “mostly in the nipple”, “mostly in the breast” or “equally in both nipple and breast”. They were asked to gauge their pain on a plastic slide-rule with numbers on one side and smiley or sad faces on the other side. The numbers ranged from 0 = no pain to 10 = unbearable pain both during and after breastfeeding. A five-grade scale was used to measure to what extent the mothers felt that their pain interfered with breastfeeding: 1 = no interference to 5 = extreme interference. The women were asked to describe the symptoms of pain they experienced in both the breast(s) and the nipple(s): radiating, non-radiating, burning, non-burning and penetrating or non-penetrating pain. Symptoms from the nipples were described as burning or dry scaling skin on the nipple and areola, vivid pink, thinning or shiny nipple and areola tissue and participants were asked to answer “yes” or “no” for each of these descriptions of nipple appearance.

At recruitment, a sample of breast milk was given by all the participants, both case and control groups. Breast milk was collected from the most painful breast in the case group and from either breast in the control group. The women used clean medical gloves to hand express approximately one millilitre of breast milk, which was then discarded. The next step was cleansing of the nipple and aereola with sterile gauze drenched in sterile sodium chloride (9 mg/ml) followed by application of an electrical breast pump to extract approximately two millilitres of breast milk. All loose components of the breast pump were sterile and disposable as were the 10 ml test tubes that were used to collect the milk. Samples from the control group were transported to the university hospital laboratory in special cold bags containing ice. Samples from the case group were immediately placed in a refrigerator at + 5 °C. All samples reached the laboratory within 30 min after sampling and were from there transported at minus 20 °C for analysis at the microbiological unit at a nearby university hospital. The samples were cultivated on Sabouraud Agar and CHROM Agar *Candida* for five days at 30 °C and analyses carried out for *Candida* spp. Results of the cultivation were given simply as “positive” or “negative” and the species type was identified. The milk samples have been retained in biobank BD47 at − 20 °C where they will remain until the results of the study have been published.

The Breastfeeding Self-Efficacy Scale (Short Form) [13] comprising 14 statements answerable on a 5-point Likert scale was administered to all participants at recruitment. The items on the scale all begin with the statement *“I can always.*. *.*” and the extremes of the 5-point scale are: 1 = not at all confident and 5 = always confident. The range of possible total scores on the BFSES -SF is 14–70.

Women in the control group received a follow-up questionnaire by email at four weeks after the first contact. They were asked if they had had symptoms connected to perceived *Candida* overgrowth during the last four weeks including breast and/or nipple pain under and after breastfeeding measured from 0 to 10 where 0 = no pain and 10 = unbearable pain. They were also asked to indicate whether they continued to breastfeed; exclusively, partially or not at all. The participants in the case group answered a telephone questionnaire at four weeks after the first contact. The questions were the same as those posed to the control group.

### Statistical analyses

Data were analysed using SPSS version 22. The case and control groups were compared for background variables; mothers’ age, educational level, parity, history of diabetes, *Candida* infection in pregnancy and previous breastfeeding, use of antibiotics during and after birth and infants age. The case group was divided into those whose breast milk showed a growth of *Candida albicans* and those whose milk did not show a growth of *Candida albicans*. These two groups were then compared for differences in type of breast symptoms. Primiparous and multiparous mothers in the case and control groups were compared for scores for items on the BSES –SF and for total scores. For comparisons of continuous variables, the student’s t-test was used and for all other variables Pearson’s Chi-2 test (Fisher’s exact test, where appropriate) were used. The primary hypothesis was tested using Fisher’s exact test.

## Results

Data from 70 currently breastfeeding women were included in the analysis: 35 cases and 35 controls. There were two significant differences in background data between the cases and controls: statistically fewer women in the control group were primiparous and as a result of this, fewer women in the case group had previous experience of breastfeeding (Table 1).

**Table 1:** - Comparison of background variables between the case and control groups

	Case group *n* 35 (%)	Control group *n* 35 (%)	*p* - value
Mother’s age in years in mean (range)	30 (19–42)	33 (23–42)	0.07
Highest education level:			
Primary school	1	0	
High school	12 (34.3)	10 (28.6)	0.73
University / College	22 (62.9)	25 (71.5)	0.61
Infant age at 1st contact in weeks in median (range)	5 (2–36)	9 (3–24)	0.20
Parity: primipara	20 (57.1)	9 (25.7)	0.02*
Birth: vaginal	31 (88.6)	32 (91.4)	1.00
Type 1 diabetes	0	0	
Gestational diabetes, last pregnancy	1 (2.9)	0	
Vaginal Candida infection during pregnancy	5 (14.3)	3 (8.6)	1.00
Antibiotics during birth	7 (20.0)	6 (17.0)	1.00
Antibiotics after birth	5 (14.3)	4 (11.4)	1.00
Previous breastfeeding	15 (42.9)	26 (74.3)	0.02*
History of Candida in breasts during previous breastfeeding	5 (14.3)	2 (5.7)	0.43

Student’s t-test and Pearson’s Chi-2 test

*Statistically significant at < 0.05 level

Results of the breast milk cultivation showed growth of *Candida albicans* but no growth of any other *Candida* spp. The hypothesis that breastfeeding women with symptoms commonly associated with *Candida albicans* infection will have a growth of *Candida albicans* in their breast milk significantly more often than women without these symptoms was confirmed (*p* <  0.01): none of the women in the control group and eight of the 35 women in the case group showed a growth of *Candida albicans* in their breast milk. The rate of *Candida albicans* infection for the whole study group (*n* = 70) was 11.4%.

Women in the case group had suffered symptoms for a mean of 15 days before consulting healthcare professionals. Comparison of symptoms between those in the case group who had and did not have a growth of *Candida albicans* in their breast milk showed no statistically significant difference for any of the symptoms (Table 2). Comparison of breast pain during and after breastfeeding, measured at the first contact with the clinic showed no statistically significant difference between those with (mean 8.0, SD 1.8) or without (mean 6.6, SD 2.3) a positive culture for *Candida albicans* (*t* = 1.62, *p* = 0.12).

**Table 2:** - Comparisons of mothers’ symptoms and pediatricians’ diagnosis of infant candidiasis between cases with and without positive cultivation of *Candida albicans*

Variables with positive response	Whole case group *n* = 35 (%)	Growth of C. albicans in breast milk *n* = 8 (%)	*p* – value
Bright pink skin on the nipples/areolas	30 (85)	6 (75)	0.17
Flaky skin on the nipple/areolas	9 (26)	2 (25)	1.00
Thin shiny skin on the nipples/areolas	14 (40)	3 (4)	1.00
Tiny fissures on the nipples	20 (57)	6 (75)	1.00
Pain in the nipples while breastfeeding	33 (94)	8 (100)	0.81
Pain in the nipples after breastfeeding	30 (86)	7 (88)	0.39
Pain in breasts during/after breastfeeding	35 (100)	8 (100)	0.22
Paediatrician’s diagnosis of infant’s oral Candidiasis	29 (83)	8 (100)	0.30

Pearson’s Chi-2 test

At the four week telephone questionnaire follow-up, 13 women in the case group were still experiencing pain. Of these three had shown a positive culture for *Candida albicans* and ten had a negative culture. A total of five (14%) women in the case group (three with and two without positive culture results) had ceased breastfeeding because of continued problems. Two women in the case group developed symptoms of mastitis two weeks after their first contact with the clinic. Neither of these women showed a growth of *Candida albicans* in their breast milk. None of the women in the control group developed symptoms of *Candida albicans* overgrowth, reported breast pain or developed symptoms of mastitis at the four-week follow-up. They all continued to breastfeed either exclusively or partially.

The short form of The Breastfeeding Self-Efficacy Scale (BSES-SF) was administrated for every participant. There were no statistically significant differences between primiparous cases and controls for breastfeeding self-efficacy scores: neither for total BSES-SF nor individual items (Table 3). There were statistically significant differences for six items on the BSES-SF between multiparous cases and controls, all of which showed significantly higher scores for the control group. The items pertained to milk volume, coping with breastfeeding, breastfeeding without formula, feelings of satisfaction with breastfeeding, motivation to continue breastfeeding and general satisfaction with the breastfeeding experience. There was also a statistically significant difference for total BSES scores when multiparous cases and controls were compared: multiparous women in the control group had higher scores for breastfeeding self-efficacy, *p* <  0.01 (Table 4).

**Table 3:** - Comparison between primiparous cases *(n* 20) and controls (*n* 9) for breastfeeding self-efficacy scores

BSES-SF Items (each item is preceded with the words “I can always. ..”)	Mean Score Cases	Mean Score Controls	*p* – value
1. Determine that my baby is getting enough milk	3.85	4.67	0.73
2. Successfully cope with breastfeeding like other challenging tasks	3.75	4.40	0.58
3. Breastfeed my baby without using formula as a supplement	3.75	4.56	0.14
4. Ensure that my baby is properly latched on the whole feeding	3.75	4.22	0.22
5. Manage the breastfeeding situation to my satisfaction	3.30	4.11	0.10
6. Manage to breastfeed even if my baby is crying	4.00	3.89	0.82
7. Keep wanting to breastfeed	4.45	4.78	0.28
8. Comfortably breastfeed with my family members present	4.10	4.44	0.51
9. Be satisfied with my breastfeeding experience	3.55	3.78	0.63
10. Deal with the fact that breastfeeding can be time-consuming	4.35	4.00	0.25
11. Finish feeding my baby on one breast before switching to the other breast	4.05	4.33	0.53
12. Continue to breastfeed my baby for every feeding	4.25	4.44	0.65
13. Manage to keep up with my baby’s breastfeeding demands	3.80	4.22	0.42
14. Tell when the baby is finished breastfeeding	3.65	3.67	0.98
Total BSES-SF scores	50.60 (SD 11.0)	54.44 (SD 8.0)	0.37

Student’s t-test

**Table 4:** - Comparison between multiparous cases *(n* 15) and controls (*n* 26) for breastfeeding self-efficacy scores

BSES Items (each item is preceded with the words “I can always. ..”)	Mean Score Cases	Mean Score Controls	*p* – value
1. Determine that my baby is getting enough milk	3.80	4.40	0.04*
2. Successfully cope with breastfeeding like other challenging tasks	4.00	4.54	0.04*
3. Breastfeed my baby without using formula as a supplement	3.80	4.65	0.04*
4. Ensure that my baby is properly latched on the whole feeding	4.00	4.50	0.10
5. Manage the breastfeeding situation to my satisfaction	3.13	4.31	< 0.01*
6. Manage to breastfeed even if my baby is crying	4.13	4.54	0.14
7. Keep wanting to breastfeed	4.20	4.77	0.03*
8. Comfortably breastfeed with my family members present	4.70	4.62	0.83
9. Be satisfied with my breastfeeding experience	3.30	4.54	< 0.01*
10. Deal with the fact that breastfeeding can be time-consuming	4.20	4.30	0.81
11. Finish feeding my baby on one breast before switching to the other breast	4.50	4.50	0.90
12. Continue to breastfeed my baby for every feeding	4.40	4.50	0.78
13. Manage to keep up with my baby’s breastfeeding demands	4.00	4.60	0.60
14. Tell when the baby is finished breastfeeding	4.40	4.40	0.94
Total BSES-SF scores	52.70 (SD 8.4)	58.70 (SD 5.0)	< 0.01*

*statistically significant at 0.05 level

Student’s t-test

## Discussion

Although the primary hypothesis in this study was confirmed, it is important to acknowledge that *Candida albicans* was isolated from the breast milk of only 8 of 35 (23%) of the women with symptoms of deep breast pain with or without nipple/areola symptoms. Moreover, there were no statistically significant differences in clinical signs and symptoms between cases with and without growth of *Candida albicans*. These results suggest that in a clinical setting, many women presenting with symptoms that are anecdotally attributed to infection by *Candida albicans* may be given treatment for a condition that they do not have. This is an important concern for two major reasons: 1) each time the breastfeeding mother is given pharmaceutical treatment the infant is also indirectly treated and 2) over-treatment will speed up the process of microbial resistance to pharmaceuticals which will in due time also apply to the treatments used for *Candida* infections. This could have major implications for other patient groups, for example those suffering from HIV (Human Immunodeficiency Virus) and AIDS (Acquired Immune Deficiency Syndrome).

It is important for the well being of the breastfeeding dyad that clinicians can provide adequate treatment when mothers present with symptoms of breast pain that they have often been suffering for several days or even weeks. Jiménez et al. strove to uncover the etiology of sore nipples and/or painful breasts and suggested that bacteria could be the cause of deep breast pain and that breast milk sampling and subsequent microbiological analyses should be carried out in order to provide an “*etiological diagnosis”* [6]*.* There are both scientific and clinical difficulties associated with this proposition. Scientific difficulties lie in the fact that there are as yet no answers as to how the microbiome of human milk differs according to maternal genetics, dietary habits, birth mode, and environmental factors. Therefore, in clinical practice it may not be possible to say which microbial genera are pathological for the individual woman and require treatment [17]. It may not either be possible to state that one particular microbe or genera will always require treatment when found in breast milk. Each breastfeeding woman has an individual immune response to potential pathogens and it is vital that clinicians, whilst alleviating symptoms, await the woman’s innate immune response. Failure to do so will exacerbate the serious problems we face regarding overuse of antibiotics and subsequent resistance to antibiotic therapy.

Women in the present study did not show the symptoms often described in reference to mastitis; breast erythema, increased breast tension, pain, pyrexia and general malaise [18–20]. Jiménez et al. [6] suggest that suspected *Candida albicans* infection is in fact a subacute mastitis, which is a term borrowed from studies of bovine mastitis. As there is no clear scientific consensus on the definition of human lactational mastitis [20] it would seem incautious to introduce the term “subacute mastitis”: there is certainly no clear definition of what this condition might entail in humans [21]. Also, it would be difficult to prescribe treatment for a condition that has not yet been scientifically described and classified. The women in the present study had suffered pain symptoms on average for 15 days, which would seem a long period of time for a “subacute” process. Further research is called for before clinicians and scientists can accept a change in nomenclature of the type suggested by Jiménez et al. In a clinical study carried out in Sweden, 85% of women with classical symptoms of mastitis recovered without recourse to antibiotic therapy [22, 23]. It seems, in view of this, that treatment of a “subacute” condition should not be recommended as it may be of little value to the individual and might be of great detriment to the global community.

Women turn to breastfeeding clinics when they have exhausted their own problem-solving capacities. What advice and treatment then, can clinicians offer women who are on the verge of terminating breastfeeding? Women suffering from deep breast pain need to know that their symptoms are taken seriously and that although the cause of their pain may not be clear, care providers will do their utmost to help alleviate the symptoms. In the clinical situation the focus must be on the identification of problems particularly related to breastfeeding technique since poor positioning and incorrect attachment of the infant to the breast are known to cause breast and nipple pain [24, 25]. A breastfeeding consultant should carry out an analysis of the breastfeeding situation including careful observation of a breastfeeding session. Assessment of nipple damage or distortion can help guide adjustment of position and attachment during a simultaneous dialog with the mother to ascertain her sensations of pain. Thorough examination of the infant’s oral anatomy may provide indications concerning the infant’s oral coordination and whether short frenulum might be an issue [26].

In the present study paediatricians made a diagnosis of oral candidiasis in 29 of the 35 infants (82.8%) of mothers in the case group. Only eight (22.8%) of the 35 case mothers in the study had a growth of *Candida albicans* in their breast milk, which is interesting since other researchers suggest that positive breast milk cultivations are a result of contamination from the infant’s mouth [3]. The clinical diagnosis of oral thrush in healthy infants is most often made by the observation of a white covering on the infant’s tongue or oral mucosa. It is possible that milk coating can be misinterpreted as *Candida* overgrowth. Moreover, the presence of *Candida albicans* in the mouths of young infants is common and may not necessarily be a sign of morbidity. A recent study from Sweden [27] reported the presence of *Candida albicans* in 10 - 15% of healthy infants during the first 12 months of life and other studies have shown a range of between 7 - 65% for the occurrence of *Candida* spp. in healthy infants [28–30]. A study from Brazil showed that non-breastfed infants had a positive cultivation of *Candida* spp. statistically more often than breastfed infants (67% vs. 35%) [30].

There was a statistically significant difference in parity (and therefore in previous breastfeeding experience) between cases and controls. It is a weakness in the study design that data collection was not stratified for primiparous and multiparous participants. Stratification for parity in the analyses of the BSES-SF may to some extent rectify this over-sight, although the study was not powered for the analysis of BSES-SF. Despite this, results of comparisons between primiparous and multiparous participants for breastfeeding self-confidence are interesting. Six statistically significant differences for items on the BSES-SF between cases and controls were all in the multiparous group: the cases scored lower on each item. The statistically significant items concerned mothers’ uncertainty about the baby receiving enough milk, which affected their ability to manage and be satisfied with the breastfeeding experience. One might expect primiparous mothers to be those with less self-confidence in breastfeeding. We reason, therefore, that it is possible that these multiparous mothers had poor previous experiences of breastfeeding and therefore entered the new breastfeeding scenario with trepidation. This in turn may be a reason why mothers in the case group turned to healthcare providers: their experiences of breast pain may in fact be an indication of their need to be supported in their breastfeeding uncertainty. Further investigation will help determine whether the BSES-SF might be a useful tool in the clinical situation when breastfeeding mothers turn to healthcare providers with diffuse symptoms of breast pain. If health professionals are familiar with the BSES-SF they will be able to identify mothers who appear to be unsure of their breastfeeding ability and provide appropriate care.

In our study the milk samples were cultured on Agar plates and it could be argued that this is an outdated method. This is however the method of analysis most commonly used in Sweden and no other method for breast milk analysis is available at the laboratory where the analyses were carried out. Moreover, it has been shown in other studies that culturing on Agar plates is an adequate method to indicate growth of *Candida* colonies [3, 4]. In a study from Germany researchers found *Candida albicans* in breast milk from women with and without the supposed symptoms of *Candida albicans* infection [31]. They compared the use of Agar plating to the use of Real Time PCR (Polymerase Chain Reaction) as analysis methods and showed no improvement in detection of *Candida* spp. with the PCR method. In contrast, many more *Candida* spp. were isolated when PCR was used than when culture was used in the CASTLE study from Australia [2]. Researchers strive to find answers that are generalizable to a larger population. The microbiota of breast milk is diverse, and immune factors found in breast milk differ geographically [16]. A deeper understanding of the microbiome of the lactating breast is required before we can answer the question of “what is normal and what is pathological”. It is possible that this question may be answerable only at the individual level rather than in the populations we create in our research studies.

## Conclusions

The answer to whether *Candida albicans* is the reason for deep breast pain during and after breastfeeding with or without skin symptoms remains elusive. Neither clinical symptoms nor microbial cultivation appear to be reliable means for making a diagnosis. Treatment with antifungal medication should not be the first line treatment for women with deep breast pain during lactation. Skilled breastfeeding consultants should offer support and help with positioning, attachment and identification of physical impediments to successful breastfeeding. They should be aware that it is possible that uncertainty in the breastfeeding situation may to some extent account for mothers’ breast symptoms. Further research should focus on the efficacy of individual professional support for breastfeeding women suffering from deep breast pain and on the usefulness of the BSES-SF in clinical practice.

## Abbreviations

AIDS: Acquired Immune Deficiency Syndrome
BFSES-SF: Breastfeeding Self Efficacy Score - Short Form
CHROM: Chromogenic media
HIV: Human Immunodeficiency Virus
IBCLC: International Board Certified Lactation Consultant
ISRCTN: International Standard Randomised Controlled Trial Number
PCR: Polymerase Chain Reaction
spp.: species
SPSS: Statistical Package for the Social Sciences
VAS: Visual Analogue Scale
